# Innovative use of mesh bolster for adult Morgagni hernia repair

**DOI:** 10.1093/jscr/rjz205

**Published:** 2019-07-03

**Authors:** Nabiel Azar, Robert Azar, Katie Robertson, Priya Gupta

**Affiliations:** 1 Providence Medical Group, General Surgery, 1510 Division St. Suite 210, Oregon City, OR, USA; 2 Department of Medical Education, College of Osteopathic Medicine of the Pacific NW, Western University of Health Sciences, 200 Mullins Drive. Lebanon, OR, USA

## Abstract

Morgagni hernia is a rare type of congenital diaphragmatic hernia caused by lack of fusion of the pleuroperitoneal membrane anteriorly leading to a defect in the foramen of Morgagni. These are rare hernias and typically present early in life. Those that do not get repaired during infancy or adolescence often present later in life with variable symptoms including obstruction, incarceration, strangulation, pulmonary symptoms, chest pain, etc. Herein we present an adult case that was found incidentally after a screening computerized tomography (CT) chest scan was done for history of smoking. There are two unique aspects to this case: first, given the large size of her hernia, her only complaint was mild shortness of breath and second, the innovative use of mesh as a suture bolster.

## INTRODUCTION

Morgagni hernia is a rare type of congenital diaphragmatic hernia caused by lack of fusion of the pleuroperitoneal membrane anteriorly leading to a defect in the costosternal trigones known as the foramen of Morgagni (parasternal hiatus) [1]. The diaphragm separates from the abdomen through the inadequate embryologic fusion of the septum transversum, pluripotent membrane, thoracic mesoderm, retroglandular, aortic, and mesogastric mesoderm [1]. These rare type of hernias typically present early in life comprising only 2% of all hernias, and of this small percentage only 5% of those cases are found in adults [2]. Herniated contents can include colon, small bowel, or omentum. Presenting symptoms are variable including obstruction, incarceration, strangulation, pulmonary symptoms, and chest pain. Some may even be asymptomatic, resulting in misdiagnosis [3]. However, once diagnosed, it is recommended that Morgagni hernias undergo repair. Approaches to repair can be through the chest or abdomen; both open and laparoscopic approaches have been reported. Lack of prompt surgical repair can result in incarceration of herniated contents [4].

## CASE REPORT

**Figure 1:**
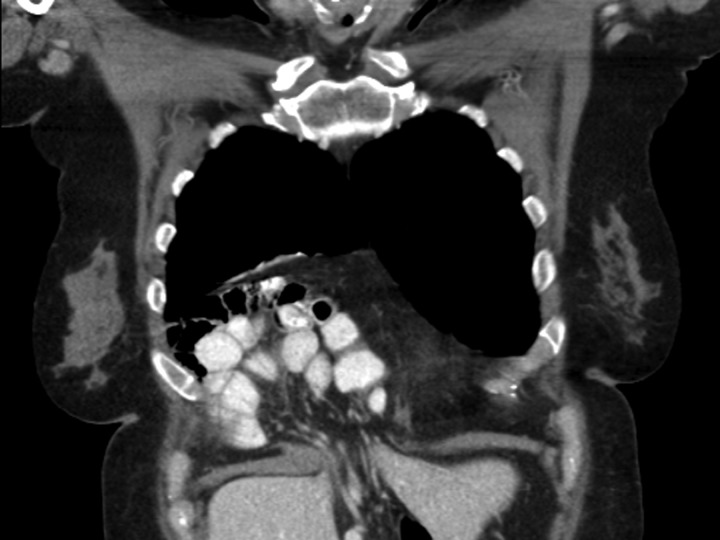
Preoperative CT scan of chest demonstrating the wide retroxyphoid defect in the diaphragm with herniated small bowel and colon into the right inferior hemithorax.

**Figure 2:**
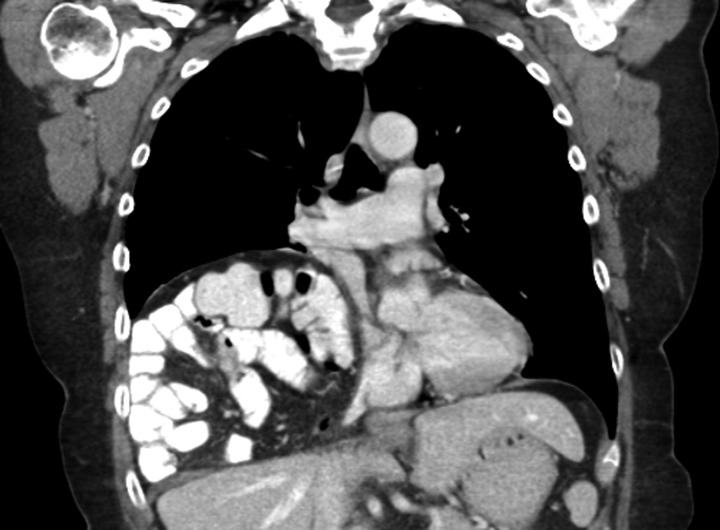
Another slice of the CT scan of the chest revealing the large size of this Morgagni hernia with multiple loops of small bowel and colon in the right inferior hemithorax.

A 69-year-old female was referred to our clinic for an incidental finding of a large Morgagni hernia found on a recent CT chest scan for lung cancer screening. Patient reported occasional shortness of breath after prolonged ambulation but denied chest pain. She did have remote history of acid reflux symptoms but nothing recently. She denied issues with prematurity or issues with development as an infant, chest trauma, or MVA history. She did complain of occasional right shoulder pain but attributed this to arthritis. Denied history of heart attack, stroke, DVT, or PE. She had a 30-pack-year smoking history but quit a year prior. She was up-to-date on her colonoscopy, current within the past year. She denied hematochezia and melena, bowel habit changes or major body weight changes as well as any current abdominal pain. On examination her vitals were within normal parameters. Heart and lungs were unremarkable. Abdominal examination was soft with normal bowel sounds and nontender. Remainder of examination was unremarkable. Laboratory values included a normal CBC and BMP. A CT chest scan had demonstrated a large retroxyphoid hernia of Morgagni involving several loops of small bowel and transverse colon located in the right inferior hemithorax (Figs 1 and 2). No evidence of acute incarceration or strangulation were noted. A detailed discussion was undertaken with the patient regarding her hernia and she was consented for a laparoscopic repair with mesh.

**Figure 3:**
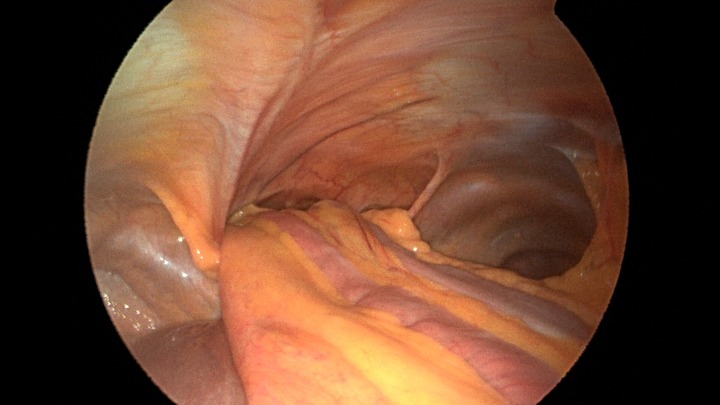
Laparoscopic view of the retroxyphoid defect in the diaphragm demonstrating multiple loops of small bowel and colon.

**Figure 4:**
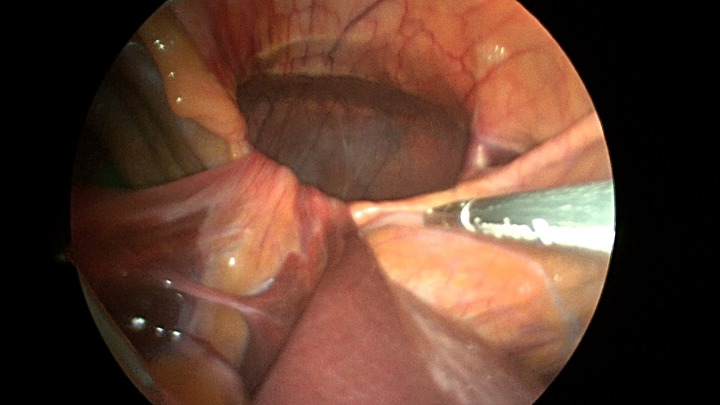
Closer view of the retroxyphoid defect demonstrating its large size with the edge of the diaphragm unattached to the undersurface of the abdominal wall.

**Figure 5:**
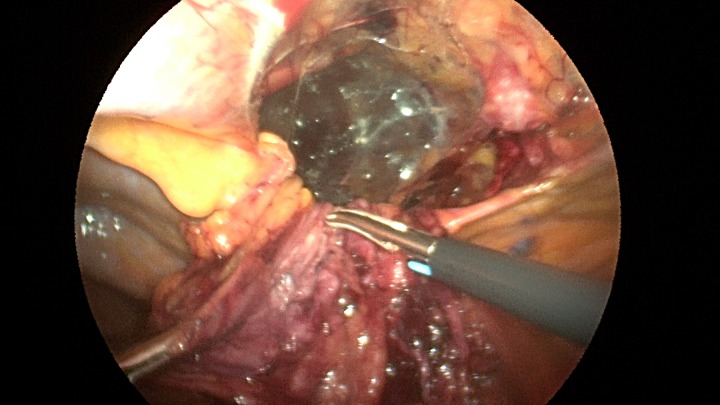
View after the parietal peritoneal hernia scan has been dissected out from the mediastinum with the sac hanging down.

**Figure 6:**
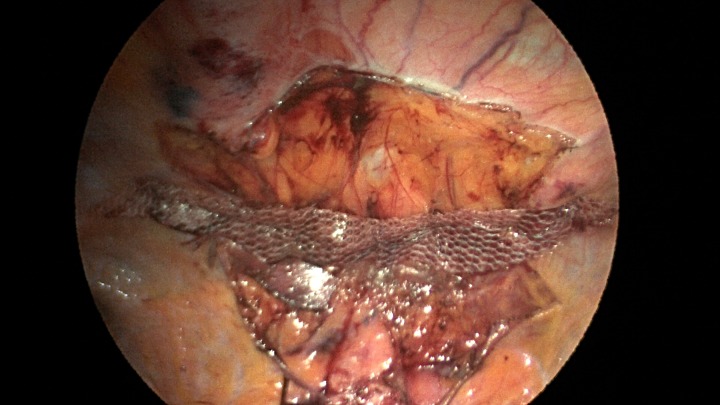
A sleeve of Parietex composite mesh cut to act as a suture bolster under the diaphragm edge to prevent the sutures from pulling through the diaphragm muscle.

Patient underwent a laparoscopic approach in lithotomy positioning with the primary surgeon working between the legs. Three working ports were used, a 12 mm port at the umbilicus and two 5 mm ports; one in the LUQ and one in the RUQ. Upon initial laparoscopy multiple loops of small bowel were progressively reduced out of the hernia sac which also included the ascending colon and part of the transverse colon (Figs 3 and 4). All the small bowel and the colon appeared viable. The redundant parietal peritoneal hernia sac was excised out of the right inferior hemithorax utilizing a LigaSure (Covidien) (Fig. 5). The falciform ligament was also taken down all the way to the diaphragm. The defect in the diaphragm measured to be approximately 9 cm by 4 cm. A section of Pariatex composite mesh was then trimmed to 2 cm in width by 9 cm in length. Three stay sutures of 0 Ethibond were placed laterally and in the middle of the mesh. This was placed into the peritoneal cavity after soaking it in vancomycin with local anesthetic. The sutures were then percutaneously brought through the diaphragm edge that was unattached to the anterior abdominal wall and then subsequently through the anterior abdominal wall. These were then tied thereby re-approximating the unattached edge of the diaphragm to the anterior abdominal wall near the xiphoid (Fig. 6). Additional 0 Ethibond sutures were placed in between these initial ones percutaneously with a suture passer.

**Figure 7:**
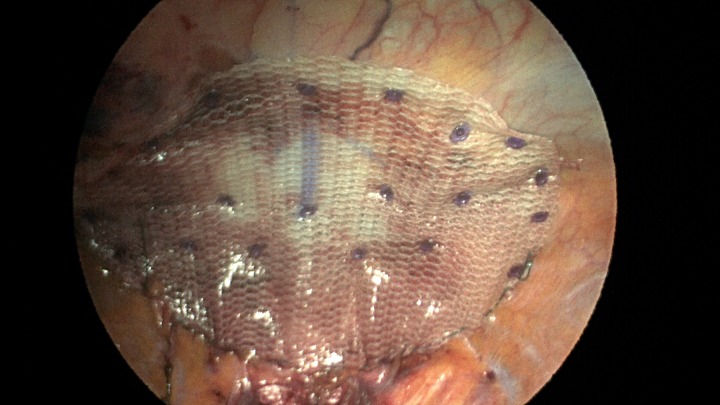
Completed view of the second main mesh piece placed with overlap of the defect and tack fixation in place.

Additionally, another Pariatex composite mesh was then trimmed to 12 cm in width by 9 cm, soaked in vancomycin with local anesthetic and then placed into the abdominal cavity. It was positioned over the area of the repair and fixed into place with absorbable tacks around its caudad edge and centrally. Along the cephalad edge it was fixed with a running V-lock absorbable suture to the diaphragm. Fibrin glue was placed along this same edge (Fig. 7). The ports were removed and incisions were closed.

**Figure 8:**
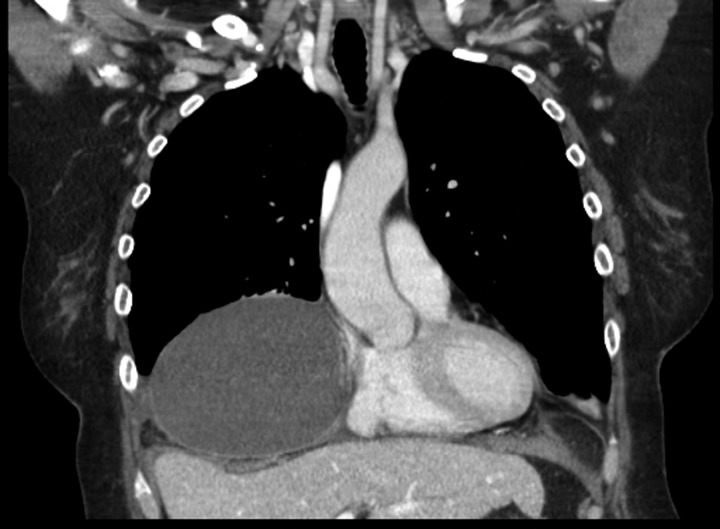
One month postoperative CT chest revealing a large seroma had formed in the area of the previous hernia.

Patient’s postoperative course progressed well. She was monitored overnight and discharged the following day. She was seen for follow-up in 2 weeks out of surgery and did quite well. She was tolerating a regular diet and having bowel movements. A month after surgery another CT scan was obtained which demonstrated a postoperative seroma in the right inferior hemithorax (Fig. 8). Currently, the patient is to be seen in a 6-month follow-up to have another CT scan at that time.

## DISCUSSION

It is not uncommon for Morgagni hernias to present with vague symptomatology. Our patient presented with respiratory symptoms such as dyspnea on exertion, but other symptoms could include, epigastric or substernal fullness, or fatigue [5]. Abdominal symptoms such as pain and constipation are typically more common. In an analysis of cases regarding Morgagni hernias in the adult population, Horton *et al.* found that almost one-third of cases are asymptomatic and subsequently discovered incidentally [6].

In this case, the patient’s herniated contents included her ascending colon, proximal transverse colon, and half of the small bowel. The patient’s peritoneum was intact resulting in a true hernia sac on the right side and the left side was not involved due to protection provided by the pericardium [7]. Interestingly, this patient had undergone a successful colonoscopy 2 years prior. It is conceivable that the colon was not herniated at that time.

A chest X-ray may demonstrate an irregular density in the cardiophrenic angle, air-fluid levels in the paracardiac region, or intestinal loops in the thorax [8]. In our patient, a previous chest X-ray dating back to 2000 showed question of a prominent pericardial fat pad. This is a common misread finding that in fact is due to this hernia.

CT scan is obtained for a specific diagnoses of Morgagni hernia in order to strategize your surgical approach. Minimally invasive laparoscopic techniques have become a popular option in the 21^st^ century due to reduced intraoperative morbidity and quicker recovery rates [9]. Our case was unique in that the novel use of a sleeve of composite mesh was used as a bolster for the Ethibond suture as opposed to felt pledgets so the suture would not pull through the diaphragm. This sleeve of mesh was separate from the main mesh that was used to cover the overall repair (see pictures).

Ultimately the operative technique will depend on the specific anatomic details and surgeon’s preference. As with many laparoscopic hernia repairs that are large and involving large dead space that may remain, postoperative seroma is expected. Our patient did develop a seroma and currently is planned to have a 6-month follow-up CT scan to check for resolution. Options of treatment, if it still remains could be percutaneous drainage or even instillation of sclerosing agents if needed. This would be treated as a refractory pleural effusion.
